# Methanol May Function as a Cross-Kingdom Signal

**DOI:** 10.1371/journal.pone.0036122

**Published:** 2012-04-26

**Authors:** Yuri L. Dorokhov, Tatiana V. Komarova, Igor V. Petrunia, Vyacheslav S. Kosorukov, Roman A. Zinovkin, Anastasia V. Shindyapina, Olga Y. Frolova, Yuri Y. Gleba

**Affiliations:** 1 A. N. Belozersky Institute of Physico-Chemical Biology, Moscow State University, Moscow, Russia; 2 N. I. Vavilov Institute of General Genetics, Russian Academy of Science, Moscow, Russia; 3 N. N. Blokhin National Cancer Research Center, Russian Academy of Medical Sciences, Moscow, Russia; 4 Nomad Bioscience GmbH, Biozentrum Halle, Halle (Saale), Germany; City of Hope National Medical Center and Beckman Research Institute, United States of America

## Abstract

Recently, we demonstrated that leaf wounding results in the synthesis of pectin methylesterase (PME), which causes the plant to release methanol into the air. Methanol emitted by a wounded plant increases the accumulation of methanol-inducible gene mRNA and enhances antibacterial resistance as well as cell-to-cell communication, which facilitates virus spreading in neighboring plants. We concluded that methanol is a signaling molecule involved in within-plant and plant-to-plant communication. Methanol is considered to be a poison in humans because of the alcohol dehydrogenase (ADH)-mediated conversion of methanol into toxic formaldehyde. However, recent data showed that methanol is a natural compound in normal, healthy humans. These data call into question whether human methanol is a metabolic waste product or whether methanol has specific function in humans.

Here, to reveal human methanol-responsive genes (MRGs), we used suppression subtractive hybridization cDNA libraries of HeLa cells lacking ADH and exposed to methanol. This design allowed us to exclude genes involved in formaldehyde and formic acid detoxification from our analysis. We identified MRGs and revealed a correlation between increases in methanol content in the plasma and changes in human leukocyte MRG mRNA levels after fresh salad consumption by volunteers. Subsequently, we showed that the methanol generated by the pectin/PME complex in the gastrointestinal tract of mice induces the up- and downregulation of brain MRG mRNA. We used an adapted Y-maze to measure the locomotor behavior of the mice while breathing wounded plant vapors in two-choice assays. We showed that mice prefer the odor of methanol to other plant volatiles and that methanol changed MRG mRNA accumulation in the mouse brain.

We hypothesize that the methanol emitted by wounded plants may have a role in plant-animal signaling. The known positive effect of plant food intake on human health suggests a role for physiological methanol in human gene regulation.

## Introduction

Plants are exposed to a different abiotic and biotic stress conditions [1]–[3]. Physical damage to a plant is a potential threat because it allows pathogen entry. The mechanical wounding of plant leaves after wind, rain, hail, or herbivore feeding is one of the first steps in pathogen infection and herbivore attack and activates signal transduction pathways and airborne signals to fend off harmful organisms. The mechanism by which these signals promote plant immunity remains elusive. In response to an attack by a pathogen and plant damage, several plant species emit volatile organic compounds (VOCs), including ethylene [4], methyl salicylate [5], methyl jasmonate [6], [7], nitric oxide [8], [9], and *cis*-3-Hexen-1-ol [10], which upregulate pathogen-related (*PR*) genes [10]–[12]. Pectin methylesterase (PME, EC: 3.1.1.11) [13] is a PR protein [14] and the first barrier of defense against invading pathogens [14]–[20] and herbivores [21], [22]. In higher plants, PME is a ubiquitous multifunctional enzymatic component of the plant cell wall. The *PME* genes encode a proPME precursor with an N-terminal extension of variable length [23]–[25]. The tobacco proPME protein contains a long N-terminal leader sequence with a transmembrane domain, which is important for PME delivery into the cell wall [25], [26]. PME participates in cell wall biogenesis during general plant growth [27]–[30], nematode infection [31], and pollen tube growth [32]–[35].

PME may be involved in the cell-to-cell movement of plant viruses [36] because it interacts with the movement protein of the *Tobacco mosaic virus* (TMV) [37], [38]. PME also efficiently enhances virus- and transgene-induced gene silencing (VIGS and TIGS) via the activation of siRNA and miRNA production [39], [40]. In the case of bacterial and fungal phytopathogens, PMEs function as virulence factors that are necessary for pathogen invasion and spreading through plant tissues [41], [42].

Pectin demethylation directed by cell wall PME is likely to be the main source of methanol on Earth [43]–[46]. Although wounding and herbivore attack increase methanol emissions [22], [47]–[49], methanol has long been assumed to be a metabolic waste product [43]–[45]. This point of view was supported by the observations that, in natural conditions, PME-generated methanol can accumulate in the intercellular air space of intact leaves at night [45] and, when the stomata open in the morning, methanol emission peaks are observed [46]. However, a study of the effects of the PME-generated methanol released from wounded plants (“emitters”) on the defensive reactions of neighboring “receiver” plants indicated that the methanol emitted by a wounded plant may function as a specific signal to enhance antibacterial resistance and to facilitate viral spread in neighboring plants [50]. To reveal plant methanol gene targets, methanol-inducible genes in methanol-treated *Nicotiana benthamiana* plants were identified. A model explaining the role of methanol in within-plant and plant-to-plant communication was suggested by these studies [50]. It has been hypothesized that methanol-inducible genes upregulation and enhanced virus reproduction is an unintended consequence of the plant's response against bacterial pathogens.

Methanol is widely available in human life. It is used in industrial production and is also present in windshield wiper fluid, antifreeze, and model airplane fuel. Methanol is colorless and has a taste and odor only subtly different from that of ethanol. In addition to alcoholic drinks and accidental poisoning, another source of methanol is aspartame, which is used as a synthetic nonnutritive sweetener [51]–[54]. In humans, the total methanol consumption from natural sources is estimated to average 10.7 mg/day [55].

Methanol itself has a low toxicity [56]. For example, animal cell cultures can tolerate high concentrations of methanol [57]–[60]. However, in mammalian organisms, methanol is metabolized by alcohol dehydrogenases (ADHs) to produce formaldehyde and formic acid and, further, to carbon dioxide and water [61], [62]. Ethanol, which is a substrate of ADHs, has a role as an antidote, so the inevitable methanol content in commercially available alcoholic drinks is not harmful for human health [63], [64]. In cases of accidental methanol poisoning, the current primary treatment is the inhibition of ADHs, preferably by ethanol and fomepizole (4-methyl-1*H*-pyrazole) [65]–[69].

More than 60 years ago, a small amount of methanol was detected unexpectedly in the breath of several normal, healthy humans using gas-liquid chromatography [70]–[72]. Subsequently, methanol was also identified in the exhaled breath of healthy volunteers using mass spectrometry [73]–[76]. The level of breath methanol was equivalent to the 0.038 mmol/L (1.22 mg/L) found in blood, which is more than 400 times lower than harmful concentrations [56]. In methanol poisonings, the usual criteria for hemodialysis and antidote therapy include a plasma methanol concentration >15.6 mmol/L (500 mg/L) [77]. The methanol content in the exhaled breath of volunteers was increased after fruit and fruit juice consumption [70], [75], suggesting the participation of pectin/PME in methanol generation [78]. The origin of endogenous methanol in humans is not yet clear, but two sources were suggested [70]. The first is human gut microbiota. Anaerobic fermentation by gut bacteria is known to produce a variety of VOCs, including all alcohols in the series from methanol to heptanol [79]. Although methanol-generating microbes have not yet been isolated from intestinal bacteria, this hypothesis should be investigated further. The second suggestion considers methanol to be a “product of some metabolic process” [70]. This hypothesis was supported by evidence that *S*-adenosyl methionine (SAM) may be transformed to methanol and S-adenosyl homocysteine in the bovine pituitary gland and other animal brain tissue [80], [81]. SAM is a universal endogenous methyl donor and is a limiting factor in various methylation reactions, including the methylation of proteins, phospholipids, DNA, RNA and other molecules, which are the basic mechanisms of epigenetic phenomena [82]–[84]. Protein carboxymethylase is highly localized in the brain and the pituitary gland of several mammalian species [85]–[87] Carboxylmethylation involves the methylation of the –COOH group of the amino acids in proteins, and the reaction is catalyzed by methyltransferases [88], resulting in the production of carboxyl methyl esters. Carboxyl methyl esters are unstable and are readily hydrolyzed in neutral and basic pH conditions or by methylesterase to produce methanol [86], [89], [90]. Interestingly, aspartame, which is a widely used synthetic non-nutritive sweetener, is a methyl ester of a dipeptide (*N*-*L*-*α*-aspartyl-*L*-phenylalanine) that is likely to convert to methanol with the participation of protein methylesterases [55].

Based on the data above, methanol is a natural compound in normal, healthy humans and mammalians. Here, we identified MRGs as methanol gene targets using forward and reverse suppression subtractive hybridization (SSH) cDNA libraries of HeLa cells that had been exposed to methanol. We showed that vegetable intake increases the methanol content in human plasma and MRG mRNA accumulation in human leukocytes. To approach the question of whether animal methanol is a metabolic waste product or whether methanol has specific function similar to the signaling function of methanol in plant life, we studied animal responses to digested and inhaled methanol. We showed that plant leaf wounding resulted in the emission of gaseous methanol, which increased methanol content in plasma of mice. Moreover, we identified MRGs as methanol gene targets and detected the up- or downregulation of MRGs in the brains of mice after breathing methanol and leaf vapors. We revealed a preference of the mice for the odor of methanol over other plant volatiles in a Y-maze setup and suggested that methanol may function as a cross-kingdom signal.

## Results

### Identification of MRGs

The experimental identification of animal MRGs includes serious challenges because of animal alcohol dehydrogenase, which is present mainly in hepatic cells and initiates methanol conversion into toxic formaldehyde and formic acid. Therefore, cell cultures lacking alcohol dehydrogenase activity had to be used to exclude genes involved in formaldehyde and formic acid detoxification from our analysis. To that end, we selected HeLa cells, which have been shown to have no ADH activity [91]–[93]. To identify MRGs, forward and reverse SSH cDNA libraries of HeLa cells exposed to methanol (75 mmol/L for 6 h) were constructed. Of the 27 differentially expressed transcripts, 5 appeared to be more affected in intact cells, and 22 transcripts appeared to be upregulated following methanol treatment. The cloned expressed sequence tags (ESTs) of only the genes that were upregulated in response to methanol treatment were chosen for sequencing. The methanol-specific upregulation of the SSH-identified genes was validated by virtual northern blot analysis hybridized with [^32^P]-labeled probes prepared from randomly selected differential clones, which were identified by differential screening. We identified and selected four of the most abundant SSH-identified genes for further analysis (Table 1). The first gene was glyceraldehyde 3-phosphate dehydrogenase (*GAPDH*), which has a role in glycolysis and nuclear functions, including transcription, RNA transport, DNA replication, and apoptosis [94]. The second gene, *hTax1* (human T-cell leukemia virus type I) binding protein 1 (*hTax1BP1*), encodes a cytoplasmic protein that inhibits TNF-induced apoptosis by mediating the anti-apoptotic activity of TNFAIP3 and that may also have a role in the pro-inflammatory cytokine IL-1 signaling cascade [95]. The third gene, human sorting nexin family member 27 (*hSNX27*), encodes a cytoplasmic protein that is involved in cellular endocytic trafficking and the T lymphocyte endocytic recycling pathway [96]. The last gene, human cyclin A2 (*hCycA2*), encodes a protein belonging to the highly conserved cyclin family, which is a key component of the cell cycle machinery, and functions as a regulator of cyclin-dependent kinases [97]. The human orthologs, *hGAPDH*, *hTax1BP1*, *hSNX27* and *hCycA2*, have highly conserved mouse counterparts: *mGAPDH* (Fig. S1), *mTax1BP1* (Fig. S2), *mSNX27* (Fig. S3), and *mCycA2* (Fig. S4). The known properties and functions of the genes identified by SSH are not related to formaldehyde and formic acid detoxification. We concluded that *hGAPDH*, *hTax1BP1*, *hSNX27* and *hCycA2* are genes that are sensitive to methanol, which we called MRGs.

**Table 1:** Methanol-induced ESTs from HeLa cells.

Functional annotation	Matching with	EST clones (n)	E-value	Gene bank accession number
Glyceraldehyde 3-phosphate dehydrogenase (GAPDH)	Homo sapiens Glyceraldehyde 3-phosphate dehydrogenase	2	3.6e-165	AY340484
Tax1(human T-cell leukemia virus type I) binding protein 1 (TaxIBP-1)	Homo sapiens cDNA FLJ95049 encoding Tax1 (human T-cell leukemia virus type I) binding protein 1	1	1e-180	AK314292
Human sorting nexin family member 27 (hSNX27)	Homo sapiens protein associated with schizophrenia and a gene encoding the same (DD149415)	1	1.4e-142	DD149415
Human cyclin A2	Homo sapiens cyclin A2, mRNA	1	1.7e-61	X51688

### Food intake influences methanol content in human plasma and MRG expression in leukocytes in healthy humans

**Figure 1:**
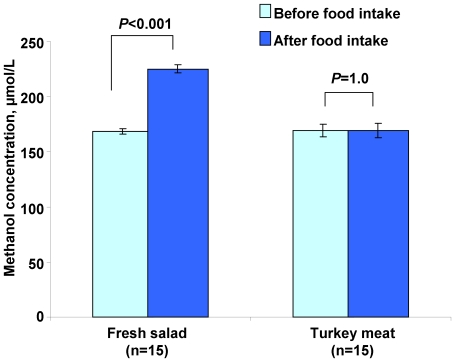
Methanol content in the plasma of volunteers 3 h after fresh salad or turkey meat intake. The data obtained in May 2011 are presented as the means ± SE. Student's *t*-test *P*-values to determine the statistical significance of the differences in methanol content before and after food intake are indicated.

To validate MRG identification and to evaluate the biological function of MRGs identified in HeLa cancer cells, we exposed cells from healthy humans to different concentrations of physiological methanol in their blood. We wanted to determine whether fresh vegetable intake serves as an additional source of human methanol. To that end, we compared the methanol content in the plasma of healthy volunteers after vegetable (Chinese cabbage, *Brassica rapa pekinensis*) and meat (turkey) intake. Tests on 15 individuals showed that fasting blood samples contained approximately 168 µmol/L methanol (Fig. 1). However, 3 h after fresh salad consumption, the methanol content increased statistically significantly up to 225 µmol/L. However, the content was at least 100 times lower than harmful methanol concentrations [56]. An analysis using an unpaired, two-tailed Student's *t*-test confirmed a statistically significant difference in methanol content between the control and fresh salad intake groups. Turkey meat intake had no effect on the methanol content in human plasma. We concluded that fresh vegetable intake increases the physiological methanol content in human blood.

**Figure 2:**
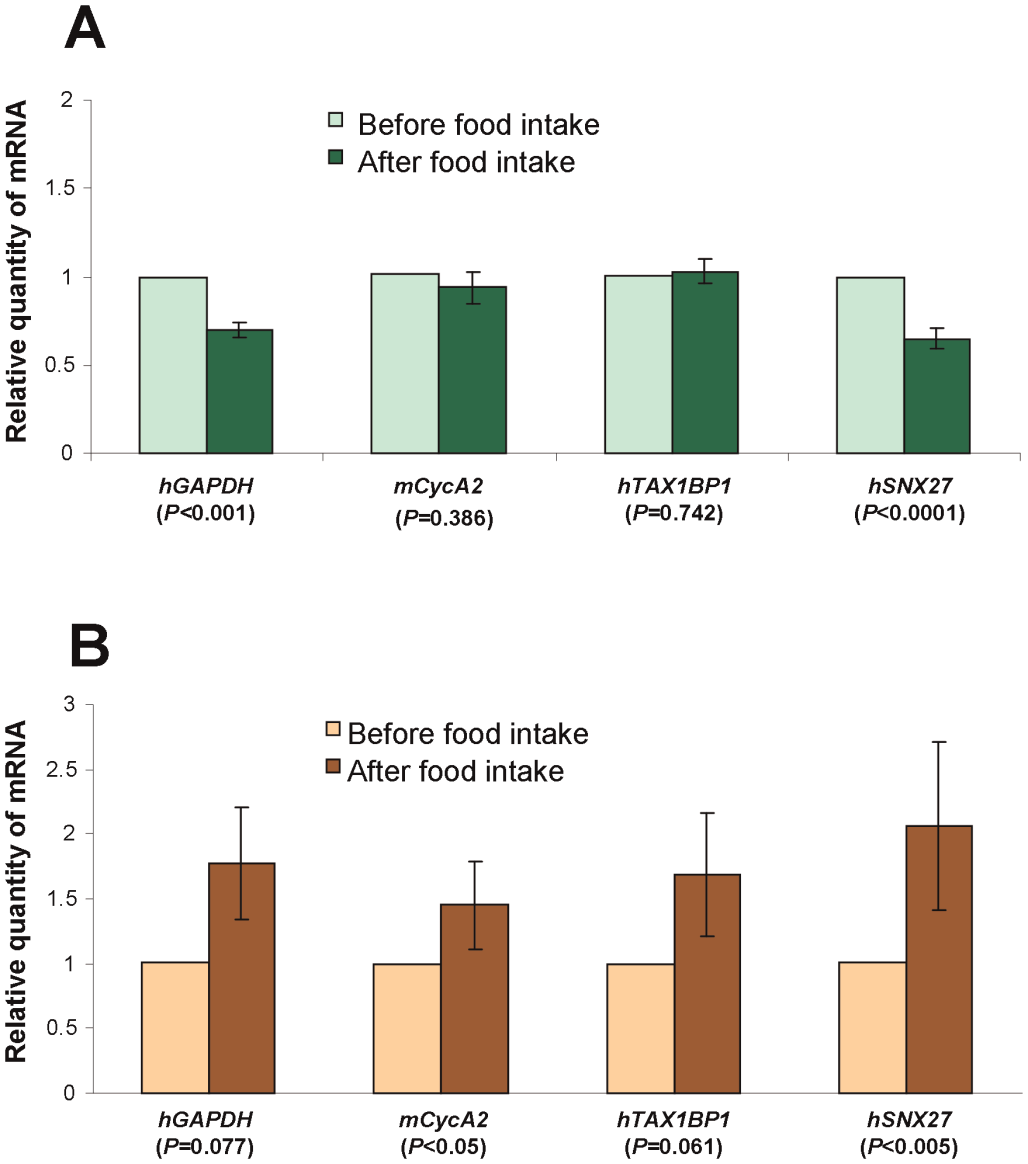
Plant-generated methanol influences gene expression in human leukocytes. (**A, B**) Influence of fresh salad (A) and turkey meat (B) intake on human blood leukocyte gene transcription as determined by qRT-PCR. The relative quantities of mRNA 3 h after food intake was normalized to the mRNA levels before food intake. Student's *t*-test *P*-values are indicated.

Subsequently, we compared the gene expression profile of the peripheral leukocytes of healthy volunteers after vegetable and meat (turkey) intake. Biologically, the increased methanol content in the blood plasma after fresh vegetable intake should lead to the up- or downregulation of human MRGs in these cells. To confirm this prediction, we studied changes in the expression patterns of the MRGs by performing quantitive real-time PCR (qRT-PCR) to determine the mRNA levels in isolated human leukocytes. Fresh salad intake resulted in a suppression of *hGAPDH* and *hSNX27* mRNA, whereas the *hTax1BP1* and *hCycA2* mRNA levels had not changed (Fig. 2A). A different MRG mRNA profile was observed after the meat diet (Fig. 2B). Turkey meat intake stimulated the accumulation of all of the MRGs. The biological meaning of this phenomenon is unclear, but gene expression in peripheral leukocytes could potentially be used as a marker of MRG expression in response to diet. We concluded that MRG mRNA accumulation in human leukocytes is sensitive to changes in the physiological methanol content in human blood plasma.

### Methanol generated in the gastrointestinal tract of mice induces the up- and downregulation of MRG mRNA in the mouse brain

**Figure 3:**
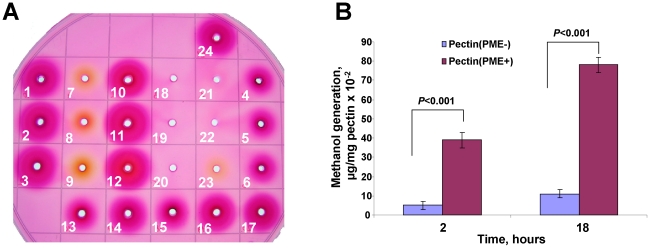
Citrus pectin preparation contains methanol-generating PME. (**A**) Detection of PME activity in plant material. Visual detection of PME activity in agarose gels containing 90% methylesterified pectin and stained by ruthenium red. The wells were loaded with citrus pectin (Nittary Pharmaceuticals, VitaLine Inc.) extract before (1–3) and after (4–6) heating (70°C, 10 min), the cell wall extract of carrot (7–9), cabbage head (*Brassica oleracea*) (10–12), sauerkraut (13–15), sauerkraut brine (16), the cell wall supernatant fraction of sauerkraut (17), the cell wall extract of dried plum (prune) (18–20) and red beet (21–23). Well #24 was loaded with pectinesterase from orange peel containing 20 nkatals PME. Plant material was extracted with Na-citrate buffer, pH 7.0, without NaCl (1, 4, 7, 10, 13, 18, 21) or with 0.15 M (2, 5, 8, 11, 14, 19, 22) or 1 M (3, 6, 9, 12, 15, 16, 17, 20, 23, 24,) NaCl. (B) citrus pectin(PME+) generates methanol. The methanol content in the pectin suspension in water after a 2-h and an 18-h incubation at 28°C. The data represent five independent experiments, and the standard error bars are indicated. Student's *t*-test *P*-values are indicated.

To support our data regarding MRG responsiveness to increases in physiological methanol, we used an experimental mouse model. In natural conditions, the pectin/PME complex is a source of methanol. To approach the question of whether ingesting pectin/PME complex-containing food generates methanol in the mouse gastrointestinal tract, we first tested PME activity in different plant preparations. To that end, we used a gel diffusion assay based on the increased binding of ruthenium red to pectin as the number of methyl esters attached to the pectin decreases [36], [98]. The diameters of the stained zones decreased with an increasing percentage of pectin esterification, which allowed for the quantification of PME activity. Figure 3A shows that extracts of citrus pectin (Nittary Pharmaceuticals, VitaLine, Inc.) contained high PME activity (∼13 nkatals/mg protein) (wells #1–3), and this activity was significantly lower after heating (70°C, 10 min) (wells #4–6). Significant PME activity (∼20 nkatals/mg protein) was revealed in the cell wall fractions of fresh cabbage heads (*Brassica oleracea*) (wells #10–12). Sauerkraut (wells #13–15), the cell wall supernatant fraction of sauerkraut (well #17), and even sauerkraut brine (#16) retained PME activity. On the other hand, the extracts of carrot (wells #7–9), dried plum (prune) (wells #18–20), and red beet (wells #21–23) did not have PME activity. Because the *B. oleracea* PME activity varied from sample to sample, we used the VitaLine citrus pectin preparation (lot Z1437) for additional studies. Next, gas chromatography was used to analyze the capacity of citrus pectin(PME+) for generating methanol. Figure 3B shows that the incubation of a water suspension of pectin(PME+) at 28°C resulted in methanol production, while pectin (cat. # P9135, Sigma), which is designated as pectin(PME−), demonstrated negligible methanol generation activity. An analysis using an unpaired, two-tailed Student's *t*-test confirmed a statistically significant difference in methanol production between the control and the pectin(PME+) sample. We concluded that pectin(PME+) was active in methanol production *in vitro*.

**Figure 4:**
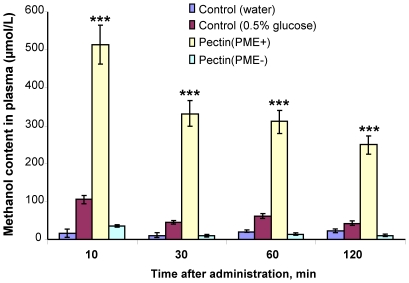
Methanol content in mouse serum 2 h after direct administration of pectin into the stomach. The mice were randomly divided into groups of ten. Each mouse in the treatment groups received 20 mg of pectin(PME+) or pectin(PME−) directly into the stomach by gavage. After 10, 30, 60 and 120 min, a blood sample was obtained and analyzed for methanol content by gas chromatography. The control groups received water or 0.5% glucose solution. The data represent five independent experiments, and standard error bars are indicated. ***, *P*<0.001 (Student's *t*-test).

To approach the question of whether the consumed vegetable (pectin/PME complex) is a source of methanol in mouse blood, we administered pectin(PME+) via a feeding tube into the stomach of mice and monitored the appearance of methanol in the blood stream by gas chromatography. The control group received a 0.5% glucose solution, pectin(PME−) or water. The methanol content in the mouse plasma increased drastically 10 min after pectin(PME+) administration then dropped slowly (Fig. 4). Methanol ingestion increased its content in plasma for 1 h and decreased to background level 2 h after injection into the stomach (Fig. S5). Pectin(PME−) resulted in a small increase in methanol content only 10 min after administration, while the water control had no influence during the course of observation. We concluded that the pectin/PME complex generated methanol in the gastrointestinal tract of mice.

**Figure 5:**
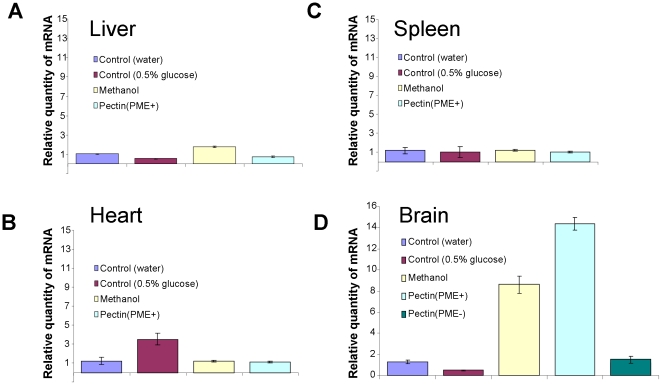
Relative quantity of *mGAPDH* mRNA in mouse organs after methanol and pectin(PME+) ingestion as determined by qRT-PCR: A – liver, B – heart, C – spleen and D – brain.

**Figure 6:**
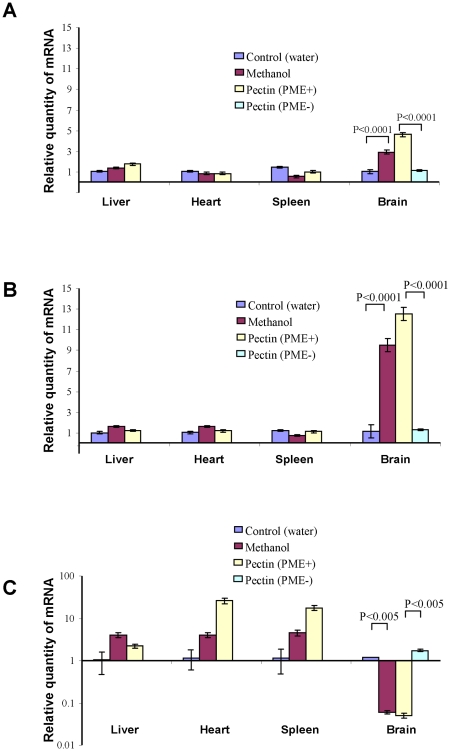
Relative quantity of (A) *mTax1BP1*, (B) *mSNX2*7 and (C) *mCycA2* mRNA in mouse organs as determined by qRT-PCR after methanol and pectin(PME+) ingestion. The *mCycA2* data (C) are plotted on a semi-logarithmic scale. The standard errors and Student's *t*-test *P*-values are indicated.

We validated the changes in expression of the mouse counterpart SSH-selected genes by performing qRT-PCR determination of mRNA levels in different organs in mice after the ingestion of methanol-producing pectin containing PME [pectin(PME+)]. The expression patterns of the four selected genes were studied in the organs of the mice 2 h after methanol and pectin(PME+) ingestion. *mGAPDH* gene expression drastically increased in the brains (Fig. 5D) of methanol- and pectin(PME+)-fed mice, whereas no increases in gene expression levels were observed in the livers (Fig. 5A), hearts (Fig. 5B) and spleens (Fig. 5C) of these mice compared to those treated with water. Similar to *mGAPDH*, an analysis of the *mTax1BP1* (Fig. 6A) and *mSNX27* (Fig. 6B) genes showed that expression was increased in mouse brains after methanol and pectin(PME+) ingestion. *mCycA2* expression was dependent on methanol and pectin(PME+) ingestion but had a more complicated profile (Fig. 6C). The accumulation of this mRNA in the livers, hearts, and spleens increased, while in the brain it was drastically suppressed. We concluded that methanol generated in the mouse gastrointestinal tract can regulate MRG mRNA accumulation in the brain.

### Methanol emitted by wounded plants directs mouse locomotor behavior during exploration

**Figure 7:**
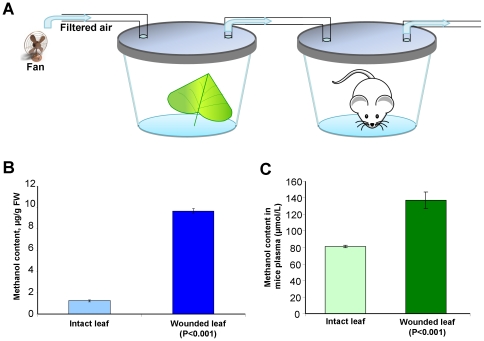
Inhalation of vapors from wounded leaves or methanol increases methanol content in mouse blood. (A) Experimental set-up for the inhalation of vapors from intact and wounded leaves by mice. A 5-l hermetically sealed vessel with five female mice was attached to a 150-ml flow-through jar supplied with filtered air (at a rate of 83.3 ml/min) and containing intact or wounded leaves from the *B. rapa pekinensis* (approximately 1 g). (B) Methanol emission by wounded leaves. *B. rapa* leaves were rubbed with Celite, and leaf samples (approximately 1 g) were loaded into hermetically sealed plastic jars with a drop of water (300 µl). After incubation for 180 min, the leaves were removed, and the methanol in the water drop was measured by gas chromatography. The standard errors and Student's *t*-test *P*-value are indicated. (C) Methanol content in the blood of female mice after a 90-min exposure to air with vapors from intact and wounded leaves. The standard errors and Student's *t*-test *P*-value are indicated.

**Figure 8:**
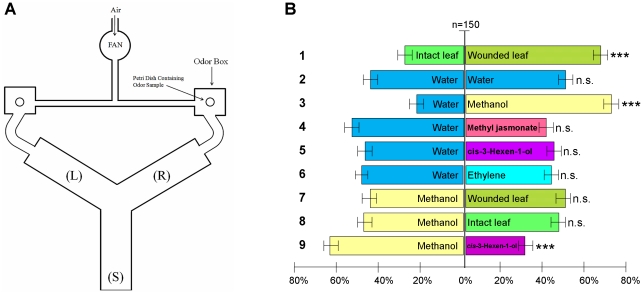
Mice prefer the odor of methanol and volatiles from damaged *B. rapa* leaves to odors from undamaged leaves. (**A**) Experimental set-up for the two-choice test based on a Y-maze. Air drawn by a fan through a tube is conducted through the left and right odor compartments. The air currents then pass to the left (L) and right (R) arms of the maze, which was fitted with a starting compartment (S). (**B**) Response of mice to different VOCs, vapors from wounded leaves and methanol compared to intact leaves and water-soaked cotton wool in the two-choice set-up. In total, 150 mice were tested per combination. The figure shows the percentages of mice selecting the odor source. Asterisks indicate a statistically significant difference within a choice. Arabic figures (on the left) designate the bar orders. χ^2−^test: *** *P*<0.01, n.s., not significantly different.

Earlier, we showed that gaseous methanol emitted by wounded plants can serve as a signal to induce defense reactions in neighbors [50]. To address the question of whether methanol functions as a signaling molecule in plant-animal communication, we studied the behavior of mice following the inhalation of vapors from wounded leaves. To that end, we used *B. rapa pekinensis* leaves rubbed with an aqueous suspension of Celite as an abrasive and loaded these leaves into a container with a flow-through setup in which the mice breathed the vapors from the damaged leaves (Fig. 7A). Leaf wounding resulted in a 10-fold increase in methanol emission (Fig. 7B) and increased the methanol content in the blood plasma samples from the mice (Fig. 7C). Subsequently, we used an adapted Y-maze (Fig. 8A) and measured the locomotor behavior of the mice during exploration in two-choice assays. We recorded the number of visits and total time a mouse spent on each side of the Y-maze as commonly assessed in preference tests. Motions that resulted in visiting one of the odor sources in the sides (L or R) of the Y-maze and stalling there for 20–25 s were recorded as choices. Motion cessation in other parts (L, R and S) of the Y-maze in which there were no odor sources was recorded as no choice. The results demonstrated that mice preferred the odors from wounded leaves (70%, *P*<0.01, χ^2−^test) to those of intact ones (Fig. 8B, bar #1) and preferred cotton wool wetted with methanol (73%, *P*<0.01, χ^2−^test) to water (Fig. 8B, bar #3). Previously [50], our gas chromatography analysis revealed the emission of *cis*-3-Hexen-1-ol, which is a representative of green leaf volatiles (GLVs), in the headspace of wounded leaves. We tested *cis*-3-Hexen-1-ol and showed that mice did not prefer this GLV to water vapors (Fig. 8B, bar #5). Moreover, a direct comparison of methanol and *cis*-3-Hexen-1-ol revealed a mouse preference for methanol (Fig. 8B, bar #9). An analysis using the χ^2−^test confirmed a statistically significant difference in the preference of mice to methanol over *cis*-3-Hexen-1-ol. We did not detect methyl jasmonate in the headspace of wounded leaves [50]. Ethylene emission was detected, but there was no statistically significant difference in ethylene emission between the control and wounded leaves [50]. Nevertheless, we tested methyl jasmonate (Fig. 8B, bar #4) and ethylene (Fig. 8B, bar #6), and the mice did not reveal any preference for these compounds over water vapors. The mice chose equally between water vapors (Fig. 8B, bar #2). Furthermore, the mice did not prefer wounded (Fig. 8B bar #7) or intact (Fig. 8B, bar #8) *B. rapa* leaves to methanol vapors.

We concluded that the methanol emitted by plants may function as an attractant for mice.

**Figure 9:**
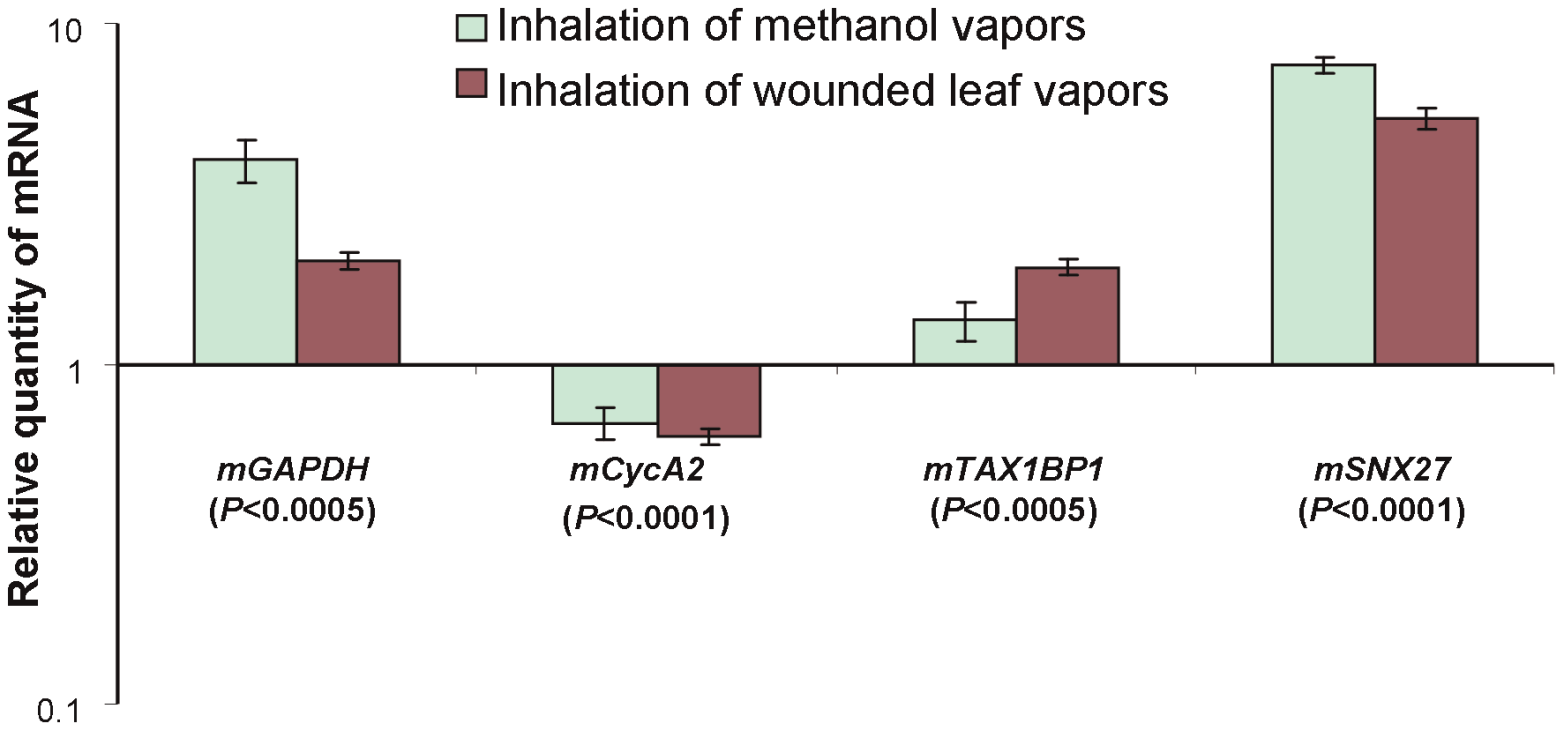
Plant-generated methanol influences gene expression in the mouse brain. Influence of vapors from wounded leaves and methanol on brain. MRG transcriptional activity as determined by qRT-PCR. The data are plotted on a semi-logarithmic scale. Relative quantities of mouse brain and leukocyte mRNA were normalized to the corresponding mRNA from control mice.

We then assessed the gene expression profile in the mouse brain after methanol inhalation. We studied the changes in the MRG expression patterns by determining mRNA levels in isolated mouse brain tissues by qRT-PCR. After the inhalation of methanol or wounded leaf vapors, the mRNA levels of *mGAPDH, mTax1BP1* and m*SNX27* increased in mouse brains, whereas *mCycA2* mRNA was suppressed drastically (Fig. 9). It is worth emphasizing that the changes in MRG mRNA levels after the inhalation of methanol or wounded leaf vapors were similar to those in the brain tissue of mice after pectin(PME+) complex ingestion (Figs. 5 and 6). We concluded that the methanol emitted by plants can be an attractant to mice and may induce the up- and downregulation of MRGs in mouse brain tissue.

## Discussion

Many plants respond to wounding from pathogen and herbivore attacks by releasing airborne volatile compounds that serve as plant defenses involved in within-plant and plant-to-plant signaling, attracting natural enemies of the herbivores and repelling other herbivores [99]. The reality of “talking trees,” which describes plants' expression of resistance mediated by VOCs from neighboring plants, is now well described [100]. The idea of “eavesdropping” has recently explained the evolutionary benefits and disadvantages for plant emitters, which mainly use VOCs for within-plant purposes [101]. Chemical signals, such as ethylene, methyl salicylate, and methyl jasmonate, induce resistance to many pathogens. Pectin and PME form a ubiquitous multifunctional enzymatic complex in the plant cell wall and generate methanol by pectin demethylation. Since 1661, when Robert Boyle [102] described methanol as a “sowrish spirit” (wood spirit) using the pyrolysis of boxwood and distillation, the function of methanol in plant and animal life has been unclear. Although emissions from volcanoes, generation from H_2_ and CO_2_ in seafloor hydrothermal systems [103]–[105] and the combustion of biomass all contribute to terrestrial atmospheric methanol, PME-mediated emissions from plants are likely the largest source of methanol in the atmosphere [43]–[46]. For a long time, gaseous methanol was considered a biochemical “waste product” [45]–[46]. Recently [50], we have studied the effects of PME-generated methanol from plants (“emitters”) on the defensive reactions of plants (“receivers”). It was shown that increased methanol emission from PME-transgenic or mechanically wounded non-transgenic plants retards the growth of the bacterial pathogen *Ralstonia solanacearum* in neighboring “receiver” plants. Antibacterial resistance was accompanied by the upregulation of genes controlling stress and cell-to-cell communication in the “receiver”. We concluded that methanol is one of the VOCs involved in within-plant and plant-to-plant signaling.

Here, we used HeLa cancer cells, which have been shown to have no ADH activity [91]–[93] and, therefore, cannot ferment methanol to produce formaldehyde. We constructed SSH cDNA libraries from HeLa cells exposed to methanol and isolated MRGs (Table 1). Moreover, we validated the MRG identification using leukocytes from healthy volunteers and showed differences in MRG expression in leukocytes following vegetable intake, which resulted in increases in methanol content (Figs. 1 and 2).

To understand the physiological role of methanol in animals, we showed that methanol generated by the pectin/PME complex (Fig. 3) may be involved in mammalian gene regulation. Methanol and pectin(PME+) ingestion resulted in the rapid appearance of methanol in mouse plasma (Fig. 4) and was accompanied by the accumulation of *mGAPDH* (Fig. 5), *mTax1BP1*, and *mSNX27* (Fig. 6) mRNA in the brain. *GAPDH* has often been referred to as a “housekeeping” gene and is used to standardize northern blots. Over the last two decades, however, a number of novel functions for GAPDH beyond glycolysis have been described, including its participation in nuclear trafficking and apoptosis [106]. Methanol-mediated *GAPDH* mRNA accumulation in the brain suggests a signaling function for methanol. This idea was supported by the simultaneous increase in the accumulation of *mTax1BP1* and *mSNX27* mRNA (Fig. 6) because recent data showed the involvement of Tax1BP1 in transcription regulation [95] and the involvement of SNX27, which is a member of the human sorting nexin family, in signal transmission [107], [108]. The effects of methanol in mouse are likely to involve many different aspects or features, including cell cycle regulation [109] as suggested by the accumulation of *mCycA2* mRNA (Fig. 6).

Here, we also showed that leaf wounding caused enhanced production of gaseous methanol and that inhaling these vapors resulted in increased methanol content in the mouse plasma (Fig. 7) and the modification of brain MRG mRNA levels (Fig. 9). We suggest that methanol can function as an attractant for mammals. In the wild, mice are primarily herbivores and eat whatever vegetation is available, including fallen seeds and fruits accumulating ethanol and methanol [110]. We propose that the methanol content in the mouse bloodstream would increase very slightly during the search for wounded plants as food but that the increase would be enough to induce the correct choice. It is worth emphasizing that short-chain alcohols, including methanol, were greatly preferred by insects and bark beetles (*Hylurgops palliatus, Tomicus piniperda*, and *Trypodendron domesticum*), while longer-chain alcohols were not attractive [111]. We suggest that mice, similar to insects, can detect this signal. In line with the hypothesis that mammals may “eavesdrop” on plant-to-plant communication signals, we showed that mice prefer the odor of methanol to other plant volatiles in a Y-maze setup (Fig. 8).

The increase in the methanol concentration following fruit and juice consumption suggests the participation of pectin/PME in methanol generation in humans. However, the high methanol content in the plasma of fasted volunteers (Fig. 1) could be explained by the participation of microbial inhabitants in endogenous methanol generation. In our experiments, glucose increased the methanol content by 5-fold compared to the water control (Fig. 4), which suggests that glucose may stimulate methanol production by the microbial inhabitants of the mouse gastrointestinal tract. The gut microbiota is likely to be an underestimated source of endogenous methanol. The second source of methanol in mammalian organism is the brain, in which intracellular metabolic processes involving SAM and protein methylesterases occur [70], [80], [81], [85], [89], [90]. Glucose-mediated increases in methanol (Fig. 4) could be explained by increased SAM production and the subsequent methanol generation. However, in our experiments, SAM ingestion did not increase the methanol content in the mouse bloodstream (data not shown), which is in accordance with the data from the experiments on volunteers [112].

We suggest that the pectin/PME complex in the diet and the generation of physiological methanol may have a positive role in human health. This hypothesis is based on indirect evidence. Studies have suggested a beneficial effect of plant food intake on human health [113], [114], the prevention of cancer [115] and cardiovascular diseases [115], [116], and the data are consistent with the proverb “an apple a day keeps the doctor away.” In addition to a substantial amount of phytochemicals, salts, minerals, and vitamins, fruits and vegetables contain a significant amounts of pectin, which might be a substrate for gastrointestinal microbial inhabitants and methanol production. Pectin has been shown to have a role in the prevention of heart disease and to exert a protective effect in hypertension and diabetes [117]–[120]. Pectin-rich foods and isolated viscous dietary fibers have demonstrated a cholesterol-lowering effect in humans [121], [122] and the reduction of atherosclerosis lesions in an animal model [123]. In addition, a substantial amount of research has suggested that fruit pectin has a role in the prevention of cancer progression and metastasis [124]–[129]. SAM is used as a dietary supplement for the treatment of many medical disorders, including depression. Interestingly, recent findings indicate a critical role for SAM in the maintenance of neuronal health, suggesting a possible role for SAM as a neuroprotective dietary supplement for Alzheimer's disease patients [130]. Although SAM intake did not result in methanol formation [112], we hypothesize a role for methanol in the maintenance of neuronal health.

**Figure 10:**
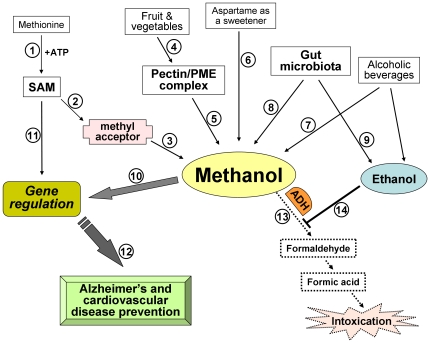
Biogenesis of physiological methanol and its relationship with ethanol. See the text for explanation.

Considering the proposed signaling function for methanol, we should estimate the toxic consequences of exogenous methanol intake and the production of endogenous methanol in humans. Methanol itself is harmless, but it is a “Trojan horse” for toxic formaldehyde [131], [132], the formation of which is catalyzed by ADH. Formaldehyde is further oxidized to formic acid by formaldehyde dehydrogenase. This conversion is very rapid, and formaldehyde has a half-life of only 1–2 min with subsequent formic acid formation [55]. Human ADH exists in multiple forms as a dimer and is encoded by at least seven different genes [133]. There are five classes (I–V) of alcohol dehydrogenase, but the primary hepatic form in humans is class 1 (ADH 1) [134]. Because ADH 1 evolved to utilized ethanol, ethanol functions as a powerful competitive inhibitor at low concentrations [135], and the enzyme has a strong preference for converting ethanol to acetaldehyde over the conversion of methanol to formaldehyde [136]. It appears that the average person may typically have endogenous ethanol in their breath [75], [76] and blood [137] that is likely produced by gut fermentation [138]. Another SIFT-MS study of breath ethanol for the same cohort of volunteers suggested that methanol and ethanol are formed in the body from different substances and/or different processes [75]. Very low levels of ethanol in the bloodstream would substantively prevent all formaldehyde production from endogenous and dietary methanol in humans. It was hypothesized [53] that the formaldehyde produced by the metabolism of dietary and endogenous methanol by ADH I would have a role in many diseases if ethanol does not act as a competitive inhibitor. Ethanol protection from formaldehyde production may explain the U-shaped curve describing the dependence on alcohol consumption and cardiovascular diseases [53], [138], [139]. Figure 10 summarizes the data on methanol and endogenous ethanol biogenesis, illustrating the putative positive role of physiological methanol and ethanol in human health. The methyl group donor, SAM, is synthesized via the catalytic activity of methionine adenosyltransferase, which transfers the adenosyl group of ATP to methionine (step 1). S-adenosylhomocysteine is formed after SAM transfers a methyl group to a methyl acceptor (step 2), such as proteins, phospholipids, DNA, RNA and other molecules, including dopamine [140]. The beneficial effects of SAM on health and its ability to reduce the progress of Alzheimer's syndrome [141]–[144] suggest its participation in gene regulation (step 11). Methyl esters, such as carboxyl methyl esters, are unstable and are readily hydrolyzed in neutral and basic pH conditions or by methylesterase to produce methanol [89], [90] (step 3). Other sources of methanol are the human diet supplying the methanol-generating pectin/PME complex from fruits and vegetables (steps 4,5), aspartame as a synthetic nonnutritive sweetener (step 6) and alcoholic beverages (step 7). The human gut microbiota is a putative source of methanol (step 8) and takes part in the generation of human endogenous ethanol (step 9). We suggest that endogenous and dietary methanol may be involved in gene regulation (step 10) and may have beneficial effects on human health (step 12). Although ADH may convert methanol into toxic products (step 13), we suggest that physiological ethanol in the bloodstream would substantively prevent all formaldehyde production from endogenous and dietary methanol in humans (step 14).

Collectively, our results indicate a cross-kingdom signaling function for methanol generated by wounded plants and in the gastrointestinal tract of mice and humans. The mechanisms through which methanol at physiologically relevant concentrations has beneficial or detrimental effects in humans remain largely undefined but include the modulation of signaling and gene regulation. Advances in the modeling and analysis of food methanol intake will extend our knowledge of methanol's role in health and disease, allowing the customization of existing and future therapeutic and prophylactic modalities.

## Materials and Methods

### Gel diffusion assay for the quantification of PME activity

PME activity in plant samples was quantified by a gel diffusion assay as described by Chen and Citovsky [36] and Downie *et al.* [98] Briefly, the tissue samples were flash-frozen in liquid nitrogen and homogenized in 1 ml of extraction buffer (0.15 M, 1 M or without NaCl, 2.5 mM phenylmethylsulfonyl fluoride, 0.1 M citrate, 0.2 M sodium phosphate, dibasic, pH 7.0). The homogenate was centrifuged at 16 000 g at 4°C, and the protein concentration of the recovered supernatant was determined by the Bradford method [145] and adjusted to the same value for all samples. These cell extracts (10 µl) were then loaded into 2-mm round wells in a 2% (w/v) agarose gel containing 0.1% of 90% esterified pectin (Sigma-Aldrich) in a Petri dish. The gels were incubated for 16 h at 28°C, rinsed with water, and stained for 45 min at room temperature with 0.05% (w/v) ruthenium red dye (Sigma-Aldrich), which stains de-esterified pectin [98]. The diameter of each stained zone was measured to the nearest 0.1 mm with calipers. The amount of PME activity in nkatals was calculated based on the standard curve of the log-transformed enzyme activity versus the stained zone diameter generated using a commercial-grade orange peel PME (Sigma-Aldrich, USA).

### Pectin administration into mice

The structure of this study and the animal experimental procedures were approved by the ethical committee of the N. N. Blokhin National Cancer Research Center, Moscow, Russia. The mice were fed a cereal-based diet, which consisted of 12.7% protein, 5.6% fat and 54.1% carbohydrate with a total fiber content of 3.7%. The diet was supplemented with a vitamin-mineral premix according to the recommendation of the American Institute of Nutrition (AIN-93M diet). The mice were randomly divided into five groups of ten mice. Each mouse in the treatment groups directly received 20 mg pectin(PME+) (Nittary Pharmaceuticals, VitaLine, Inc., USA), pectin(PME−), 200 µl 0.5% glucose solution or 200 methanol (0.375 mol/L) into the stomach by gavage. After 10, 30, 60 or 120 min, blood samples (50 µl) were isolated from the tail vein of the mice. Samples were incubated at 4°C for 2 h for cell sedimentation then an equal volume of 10% trichloroacetic acid (TCA) was added to plasma. The mixture was incubated for 20 min on ice and then centrifuged for 10 min at 16 000 g. Finally, the supernatant was analyzed for methanol content by gas chromatography.

### Two-choice experiments in mice using a Y-maze

The experimental set-up for the two-choice test was based on a Y-maze [146] with two arms, each containing Petri dishes with cotton wool soaked with 200 µl methanol, 1 µl *cis*-3-Hexen-1-ol and methyl jasmonate (odorized compartment) or 200 µl of distilled water (control compartment). Ethylene was obtained by reacting 10 M KOH with ethephon (Sigma-Aldrich, USA). Air drawn by a fan is conducted through the left and right odor compartments via a tube of which the inlet is near the input vent supplying the laboratory. The air currents then pass to the left (L) and right (R) arms of the maze, which was also fitted with a starting compartment (S). All behavioral testing took place during the light phase. The locomotor behavior of the mice during exploration in the two-choice assays was measured. We recorded the following behaviors: the number of visits and the total time a mouse spent on each side of the Y-maze. Motions that resulted in visiting one of the odor sources in the one of sides (L or R) of the Y-maze and stalling there for 20–25 s were recorded as choices. Motion cessation in parts (L, R and S) of the Y-maze other than the odor sources was recorded as no choice. The odorant was randomly distributed in the right or left arm in each test. In total, 150 mice were tested per combination. The maze was carefully washed after each test. Mice were placed separately in the starting arm at the end of the stem and then allowed to move freely for 3 min. The movements of the mice were video recorded and analyzed with a video tracking system.

### Volunteer experiments and mRNA isolation from human leukocyte

This research received approval of the Human Subjects Committee at the N. N. Blokhin National Cancer Research Center, Moscow, Russia. All participants signed Institutional Review Board-approved consent forms. The study included 15 unrelated healthy volunteers (male and female) aged 20–60 yr. A 5-ml blood sample was taken before and after the consumption of Chinese cabbage (200 g) or turkey meat (200 g). Volunteers fasted before sampling. Blood was collected in evacuated tubes containing K_3_EDTA as an anticoagulant. Four volumes of RBC lysis-buffer (0.8% NH_4_Cl, 0.2% NaHCO_3_, 0.1 mM EDTA) were added to one volume of the whole blood. The solution was incubated 10 min, and leukocytes were sedimented by centrifugation (10 min, 1000 *g*). Subsequently, the pellet was washed two times with 2 ml 1× PBS and resuspended in 100 µl 1× PBS. Total RNA was isolated from leukocytes using TriReagent (MRC, USA) according to the manufacturer's protocol. For the methanol measurements, blood samples without anticoagulant were incubated at 4°C for 2 h for cell sedimentation, and an equal volume of 10% TCA was then added to the plasma. The mixture was incubated for 20 min on ice and then centrifuged for 10 min at 16 000 g. Finally, the supernatant was analyzed for methanol content by gas chromatography.

### Methanol measurements by gas chromatography

Methanol was measured in the headspace of intact or wounded leaves in hermetically sealed jars (water-drop set-up) or in glass flow chambers using a water sample as a trap as described earlier [50]. The water-drop set-up was achieved using hermetically sealed plastic jars (150 ml) with a drop of water (300 µl). Methanol content was determined by gas chromatography on a capillary FFAP column (50 m×0.32 mm; Varian Inc., Lake Forest, CA, USA) in a Kristall 2000 gas chromatograph (Eridan, Russia). Liquid samples were measured under the following operating conditions: carrier gas, nitrogen; nitrogen flow, 30 ml/min; air flow, 400 ml/min; hydrogen flow, 40 ml/min; injected volume, 1 µl; injector temperature, 160°C; column temperature, 75°C increased at 15°C/min to 150°C; retention time, 6.5 min; and flame ionization detector temperature, 240°C.

### Construction of SSH cDNA libraries

#### RNA isolation and cDNA preparation

Total RNA was isolated from control and methanol-treated (75 mmol/L for 6 h) HeLa cells following the LiCl method. mRNA was purified from the total RNA isolated using the PolyATtract mRNA Isolation System I (Promega, USA) following the protocol supplied along with the kit. Amplified ds cDNA was prepared from methanol-treated and control RNA using a SMART approach as previously described [147]. SMART Oligo II oligonucleotide and CDS primers (Table S1) were used for first-strand cDNA synthesis. In both cases, first-strand cDNA synthesis was performed using 0.3 µg RNA in a total reaction volume of 10 µl. 1 µl of 5× diluted first-strand cDNA was then used for PCR amplification with SMART PCR primers. 18 PCR cycles (each cycle included 95°C for 7 s, 65°C for 20 s, and 72°C for 3 min) for methanol-treated and control samples were performed. SMART-amplified cDNA samples were further digested by the *Rsa* I endonuclease.

#### Subtraction procedure

Subtractive hybridization was performed using the SSH method in both directions (methanol vs. control and control vs. methanol) as described [148]. Briefly, the following procedures were performed. For each direction, two tester populations were created by ligating different suppression adapters (Adapters 1 and 2R). These tester populations were mixed with 30× driver excess (driver cDNA had no adaptors) in two separate tubes and were denatured and allowed to re-nature. After the first hybridization, the two samples were mixed and hybridized together. The subtracted cDNA was then amplified by primary and secondary PCR. For the primary PCR, 25 PCR cycles with PCR primer 1 were performed for the subtracted methanol cDNA, and 25 cycles were performed for the subtracted control cDNA. For the secondary (nested) PCR, 10 PCR cycles with Nested primers 1 and 2R were performed for both of the subtracted cDNA samples. To eliminate type II background from the SSH-generated libraries, the mirror orientation selection (MOS) method was used for both of the SSH subtracted libraries as described previously [149]. For the MOS PCR, 22 PCR cycles with the MOS PCR primer were performed for the subtracted methanol and control cDNA samples.

#### Construction of the subtracted library

The two subtracted cDNA samples enriched with differentially expressed sequences (methanol-specific and control-specific) that were obtained from the MOS PCR were used to construct the library. In each case, approximately 40 ng of the purified cDNA was cloned into the pAl16/17 vector (pUC base vector), which was then used for *E. coli* transformation. For both libraries, the white to blue colony ratio was 65∶35.

#### Differential screening of subtracted libraries

96 (one 96-well plate) randomly picked white clones from the tester methanol-specific library and 96 (one 96-well plate) randomly picked white clones from the driver control-specific library were used for differential screening. All plates were grown in 100 µl LB-Amp (75 µg/ml) media for 6 h at 37°C. One-microliter aliquots of each of the media were used for PCR amplification with pAl16/17 dir and pAl16/17 rev plasmid primers. The plates were subsequently diluted with 20% glycerol and stored at −70°C. 2 µl of each of the PCR-amplified inserts (approximately 100 µg DNA) was arrayed in a 96-well format onto duplicated nylon membranes and hybridized with P^32^-labelled methanol- and control-subtracted cDNA probes.

#### Virtual northern blot analysis

Virtual northern blot analysis was performed to confirm the differential screening results. For the virtual northern blot analysis, SMART-amplified “driver” (methanol) and “tester” (control) unsubtracted cDNAs were resolved on agarose gels and transferred to Hybond-N membranes. Membranes were hybridized with P^32^-labeled probes prepared from randomly selected differential clones that were identified by differential screening. The clones from the methanol-subtracted library and the control-subtracted library were used for virtual northern blotting. Selected plasmids were purified and sequenced using pAl16/17 dir and pAl16/17 rev plasmid primers (Table S1).

### qRT-PCR Analysis of Transcript Concentrations

RNA concentrations were determined using a Nanodrop ND-1000 spectrophotometer (Isogen Life Sciences). All RNA samples had a 260∶280 absorbance ratio between 1.9 and 2.1.

cDNA was obtained by annealing 2 µg of denatured total RNA with 0.1 µg of random hexamers and 0.1 µg of Oligo-dT. The mixture was then incubated with 200 units of Superscript II reverse transcriptase (Invitrogen, USA) for 50 min at 43°C. The qRT-PCR was performed using the iCycler iQ real-time PCR detection system (Bio-Rad, Hercules, CA, USA). For the detection of target genes, the Eva Green master mix (Syntol, Russia) was used according to the manufacturer's instructions. The thermal profile for EVA Green qRT-PCR included an initial heat-denaturing step at 95°C for 3 min and 45 cycles at 95°C for 15 s, an annealing step (Table S2) for 30 sec and 72°C for 30 sec coupled with fluorescence measurements. Following amplification, the melting curves of the PCR products were monitored from 55–95°C to determine the specificity of amplification. Each sample was run in triplicate, and a non-template control was added to each run. Target gene mRNA levels were calculated according to the equation proposed by Pfaffl [150]: EtargetΔCt target (sample-reference). PCR efficiency (E) was calculated according to the equation E = 10(−1/slope) based on the standard curves. Target gene mRNA levels were corrected using corresponding reference genes.

## Supporting Information

Figure S1 **Amino acid sequence alignment of human and mouse GAPDH (accession numbers P04406 and P16858, respectively).** Amino acid sequences were aligned using the AliBee program (http://www.genebee.msu.su/services/malign_reduced.html). Identical amino acid residues between the human and mouse proteins are marked by asterisks. Specific protein domains are underlined.
(TIF)

Figure S2 **Amino acid sequence alignment of human and mouse Tax1BP1 (accession numbers A4D196 and Q3UKC1, respectively).** Amino acid sequences were aligned using the AliBee program (http://www.genebee.msu.su/services/malign_reduced.html). Identical amino acid residues between the human and mouse proteins are marked by asterisks. Specific protein domains are underlined.
(TIF)

Figure S3 **Amino acid sequence alignment of human and mouse SNX27 (accession numbers** Q96 L92 **and Q3UHD6, respectively).** Amino acid sequences were aligned using the AliBee program (http://www.genebee.msu.su/services/malign_reduced.html). Identical amino acid residues between the human and mouse proteins are marked by asterisks. Specific protein domains are underlined.
(TIF)

Figure S4 **Amino acid sequence alignment of human and mouse CycA2 (accession numbers AAI04784.1 and NP_033958.2, respectively).** Amino acid sequences were aligned using the AliBee program (http://www.genebee.msu.su/services/malign_reduced.html). Identical amino acid residues between the human and mouse proteins are marked by asterisks.
(TIF)

Figure S5 **Methanol content in mouse serum 2 h following direct stomach administration of methanol.** The mice were randomly divided into five groups of ten. Each mouse in the treatment group received 200 µl methanol (0.375 mol/L) directly into the stomach, and 10, 30, 60, and 120 min later, blood samples were isolated and analyzed for methanol content by gas chromatography.
(TIF)

Table S1 **Oligonucleotides used for SSH.**
(DOC)

Table S2 **Oligonucleotides used for qPCR.**
(DOC)
