# Supplementary figures and images for: Methanol May Function as a Cross-Kingdom Signal
Source: PLoS One. 2012 Apr 26;7(4):e36122. doi: 10.1371/journal.pone.0036122 (PMC3338578; doi:10.1371/journal.pone.0036122)

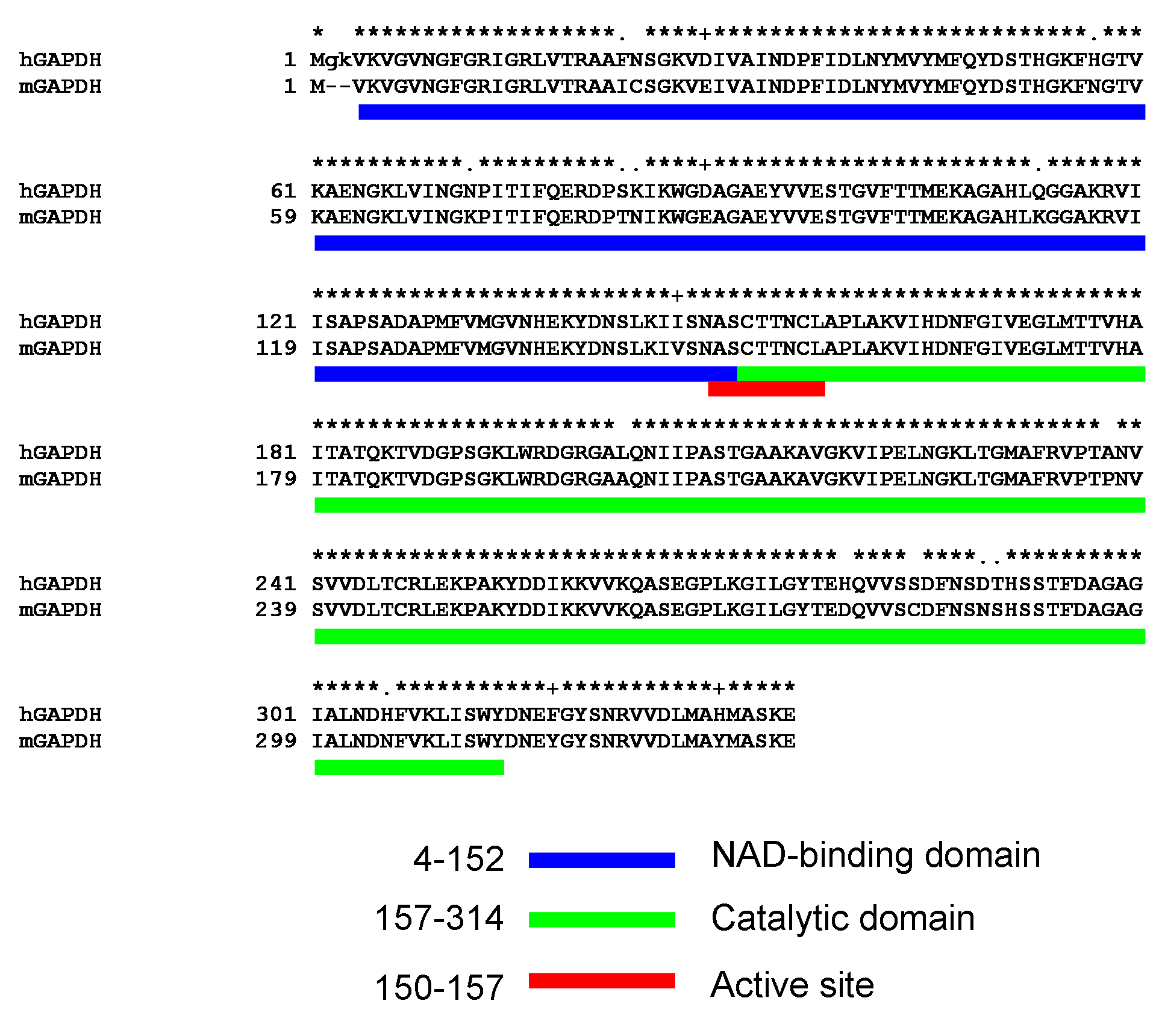

Supplement: Figure S1 — Amino acid sequence alignment of human and mouse GAPDH (accession numbers P04406 and P16858, respectively). Amino acid sequences were aligned using the AliBee program (http://www.genebee.msu.su/services/malign_reduced.html). Identical amino acid residues between the human and mouse proteins are marked by asterisks. Specific protein domains are underlined. (TIF) [file pone.0036122.s001.tif]

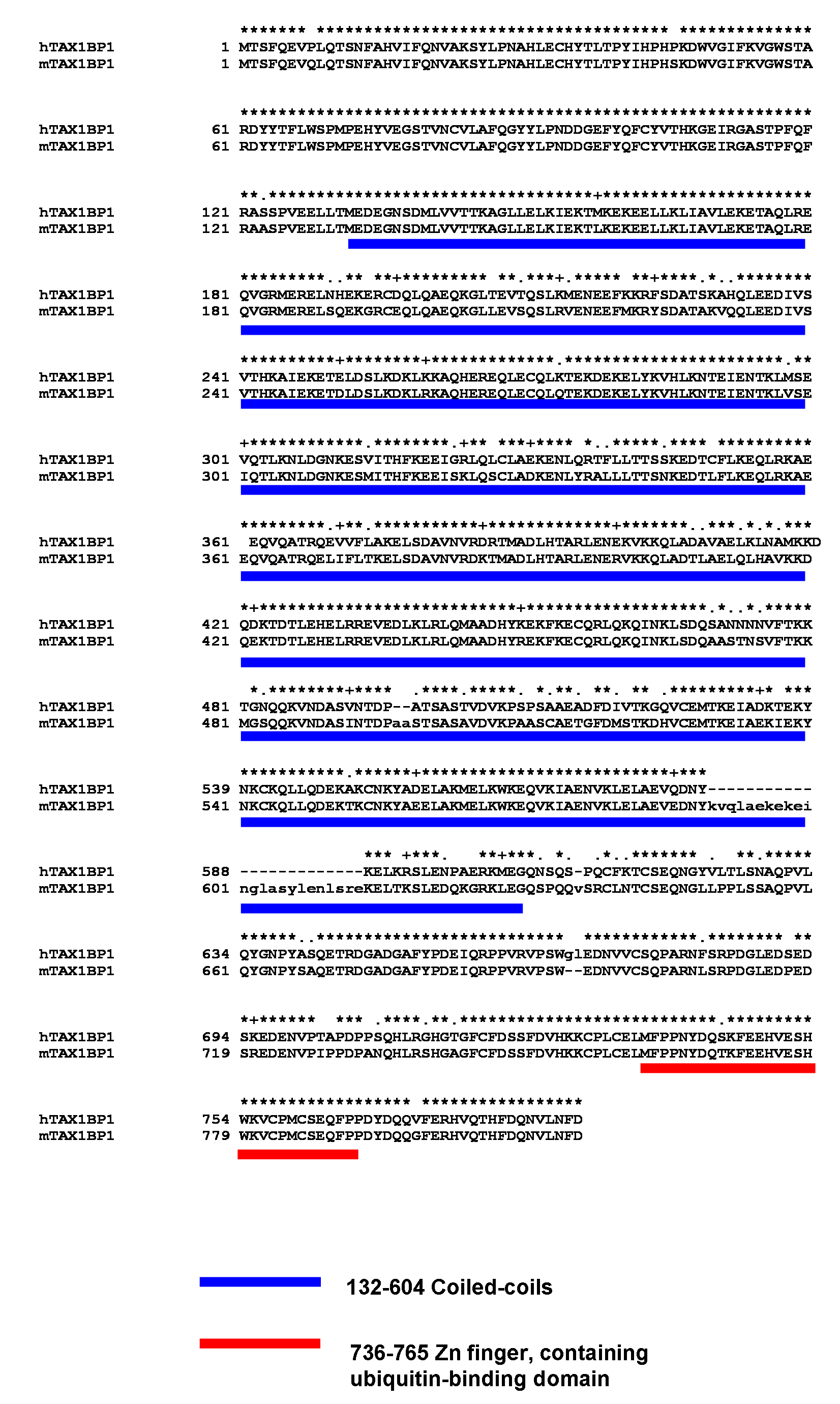

Supplement: Figure S2 — Amino acid sequence alignment of human and mouse Tax1BP1 (accession numbers A4D196 and Q3UKC1, respectively). Amino acid sequences were aligned using the AliBee program (http://www.genebee.msu.su/services/malign_reduced.html). Identical amino acid residues between the human and mouse proteins are marked by asterisks. Specific protein domains are underlined. (TIF) [file pone.0036122.s002.tif]

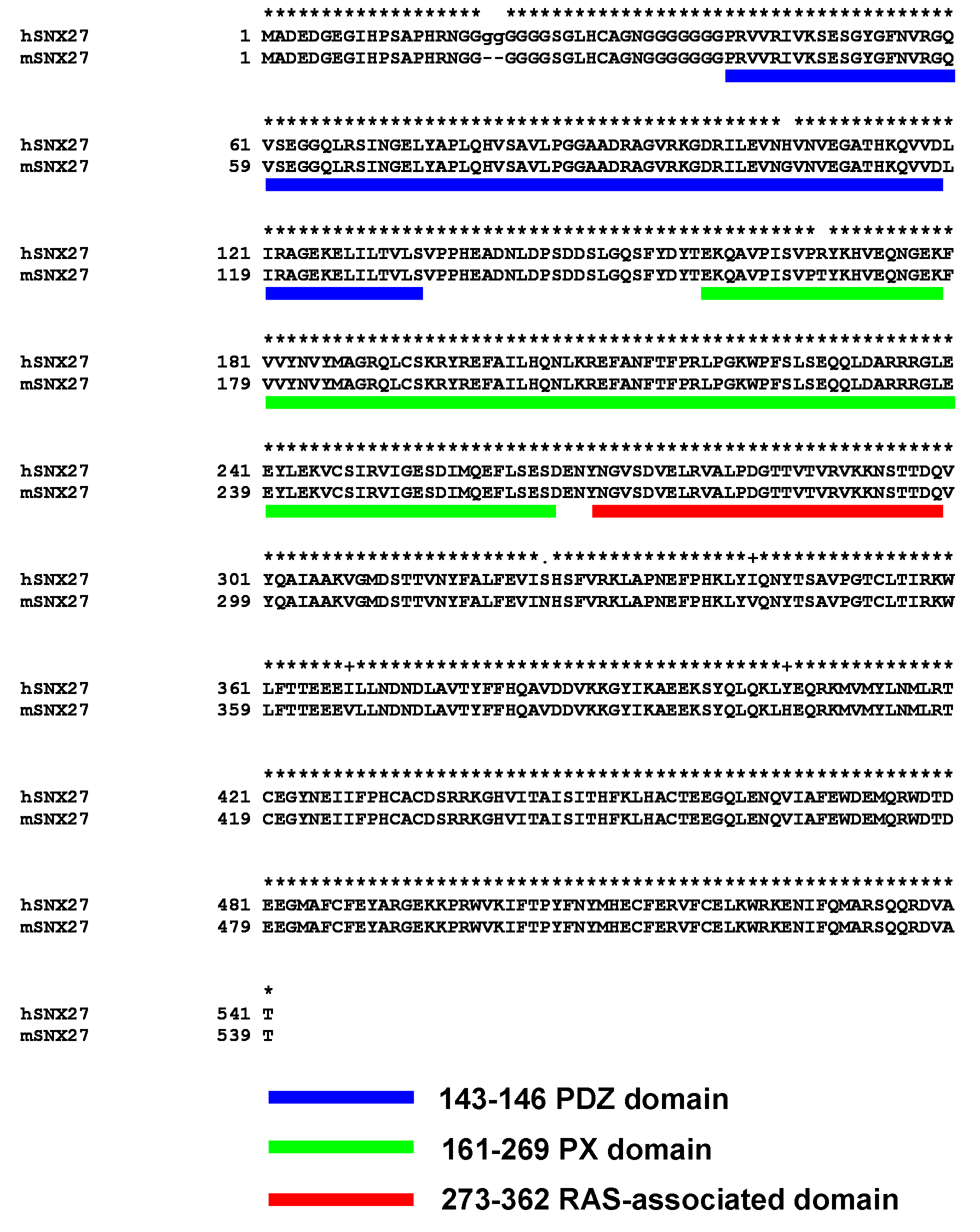

Supplement: Figure S3 — Amino acid sequence alignment of human and mouse SNX27 (accession numbers Q96 L92 and Q3UHD6, respectively). Amino acid sequences were aligned using the AliBee program (http://www.genebee.msu.su/services/malign_reduced.html). Identical amino acid residues between the human and mouse proteins are marked by asterisks. Specific protein domains are underlined. (TIF) [file pone.0036122.s003.tif]

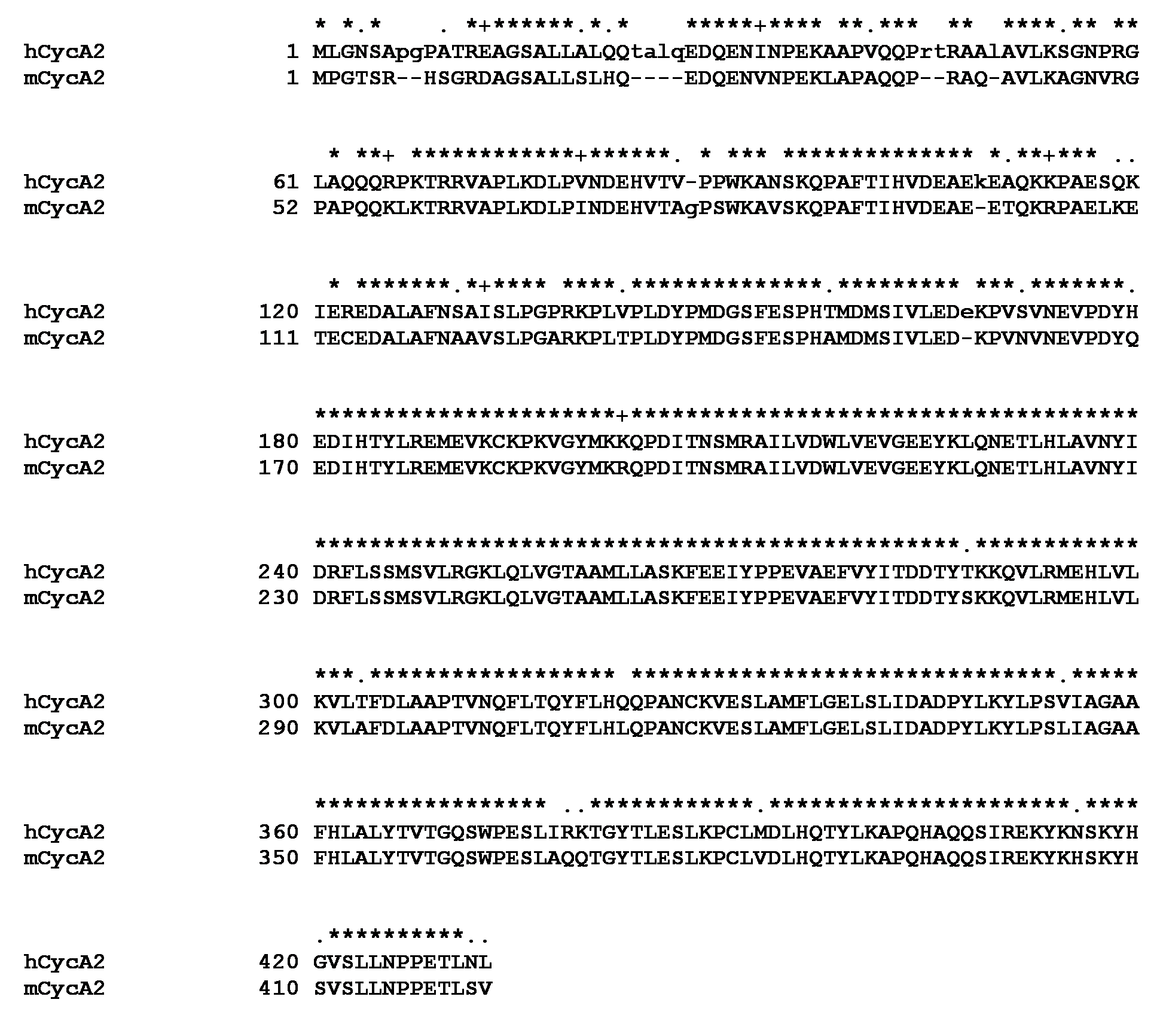

Supplement: Figure S4 — Amino acid sequence alignment of human and mouse CycA2 (accession numbers AAI04784.1 and NP_033958.2, respectively). Amino acid sequences were aligned using the AliBee program (http://www.genebee.msu.su/services/malign_reduced.html). Identical amino acid residues between the human and mouse proteins are marked by asterisks. (TIF) [file pone.0036122.s004.tif]

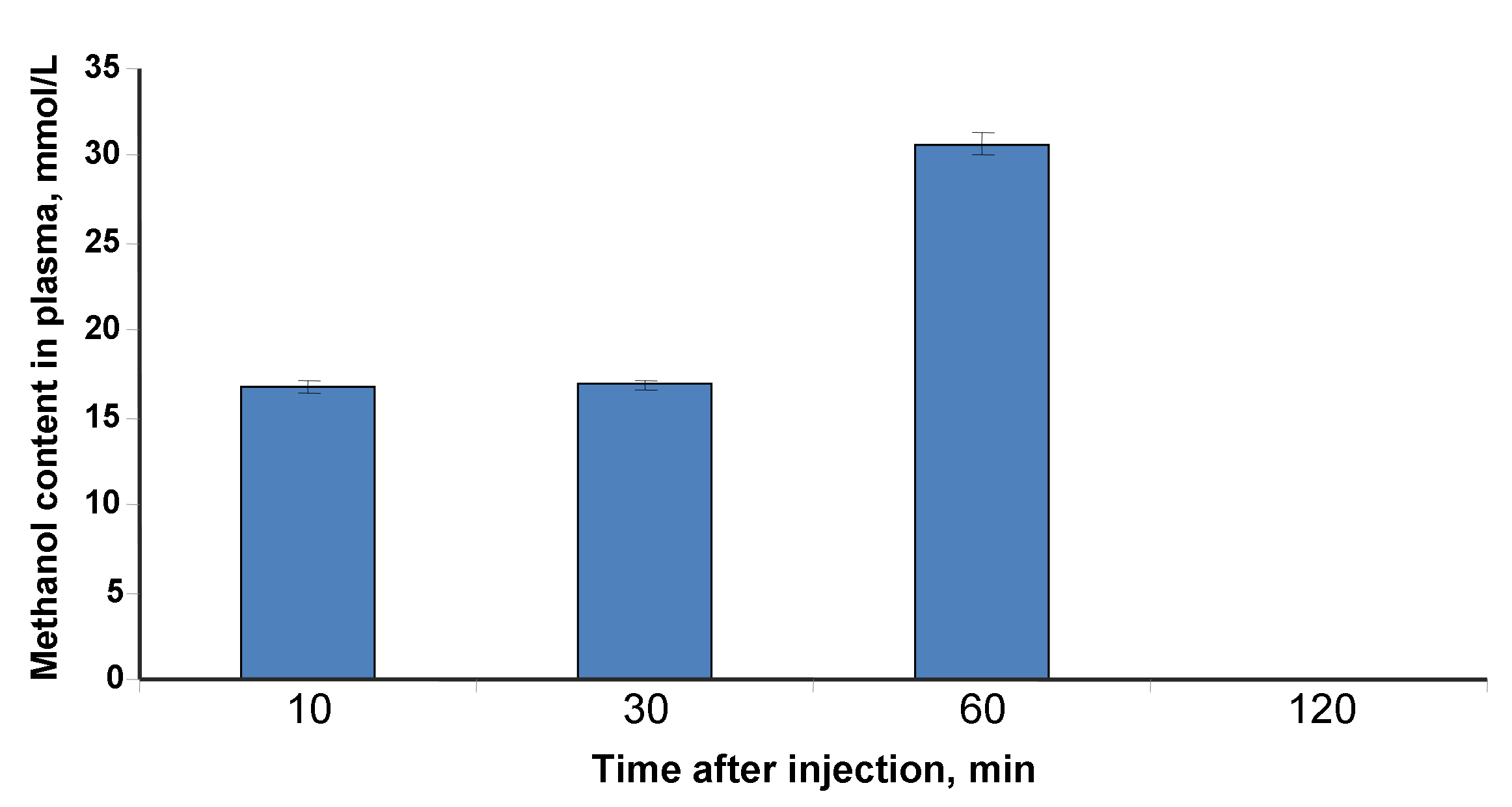

Supplement: Figure S5 — Methanol content in mouse serum 2 h following direct stomach administration of methanol. The mice were randomly divided into five groups of ten. Each mouse in the treatment group received 200 µl methanol (0.375 mol/L) directly into the stomach, and 10, 30, 60, and 120 min later, blood samples were isolated and analyzed for methanol content by gas chromatography. (TIF) [file pone.0036122.s005.tif]
