# Supplementary material for: Methanol May Function as a Cross-Kingdom Signal
Source: PLoS One. 2012 Apr 26;7(4):e36122. doi: 10.1371/journal.pone.0036122 (PMC3338578; doi:10.1371/journal.pone.0036122)
Supplement: Table S1 — Oligonucleotides used for SSH. (DOC) [file pone.0036122.s006.doc]

| SMART Oligo II A oligonucleotide | 5’-AAGCAGTGGTATCAACGCAGAGTACGCrGrGrG-3’ |
| --- | --- |
| SMART CDS primer II A | 5’-AAGCAGTGGTATCAACGCAGAGTA-d(T)30-3’ |
| SMART PCR primer II A | 5’-AAGCAGTGGTATCAACGCAGAGT-3’ |
| Adapter 1 | 5’—CTAATACGACTCACTATAGGGCTCGAGCGGCCGCCCGGGCAGGT-3’  3’-GGCCCGTCCA-5’ |
| PCR primer 1 | 5'–CTAATACGACTCACTATAGGGC-3' |
| Nested primer 1 | 5'–TCGAGCGGCCGCCCGGGCAGGT–3' |
| Adapter 2R | 5'–CTAATACGACTCACTATAGGGCAGCGTGGTCGCGGCCGAGGT–3'  3'–GCCGGCTCCA–5' |
| Nested primer 2R | 5'–AGCGTGGTCGCGGCCGAGGT–3' |
| pAl16/17 dir plasmid primer | 5'–CCAGGGTTTTCCCAGTCACGA–3' |
| pAl16/17 rev plasmid primer | 5'–CACAGGAAACAGCTATGACCA–3' |
| MOS PCR primer | 5'–GGTCGCGGCCGAGGT–3' |
