# Supplementary material for: Methanol May Function as a Cross-Kingdom Signal
Source: PLoS One. 2012 Apr 26;7(4):e36122. doi: 10.1371/journal.pone.0036122 (PMC3338578; doi:10.1371/journal.pone.0036122)
Supplement: Table S2 — Oligonucleotides used for qPCR. (DOC) [file pone.0036122.s007.doc]

| **Gene** | **Forward primer** | **Reverse primer** | **PCR product length, bp** | **Ta, ˚C** |
| --- | --- | --- | --- | --- |
| m_cyclin A2 | GAAGAGGCAACCAGACATC | GGAGGAGAGGAATCTATCAATG | 136 | 53 |
| m_Tax1bp1 | CTGTGCGAGTCCCATCTTG | TTCGTCTTCTCTGCTGTCTTC | 90 | 53 |
| m_Snx27 | TGCGAGGGCTACAATGAAATC | AAGTGCGTGATGCTGATGG | 92 | 55 |
| m_GAPDH | TCCTGGAAGATGGTGATGGGATTT | TGGTCACCAGGGCTGCTTTTA | 187 | 60 |
| m_RPL32 | GGCACCAGTCAGACCGATATG | CCTTCTCCGCACCCTGTTG | 80 | 56 |
| h_cyclin A2 | TTGGGAGAATTAAGTTTGATAGATG | GAGGTAGGTCTGGTGAAGG | 189 | 56 |
| h_Tax1bp1 | TTACCCAGATGAAATACAAAGG | GGAGCAGTAGGCACATTC | 155 | 56 |
| h_Snx27 | ACGGCATTCTCTGTGACTTC | AACCATATTCCTACTACTCCTTCC | 163 | 56 |
| h_GAPDH | TCTGGTAAAGTGGATATTGTTG | CCTGGAAGATGGTGATGG | 187 | 54 |
| h_RPL32 | CATCTCCTTCTCGGCATCA | AACCCTGTTGTCAATGCCTC | 152 | 56 |
